# Impact of COVID-19 Vaccine Misinformation on Social Media Virality: Content Analysis of Message Themes and Writing Strategies

**DOI:** 10.2196/37806

**Published:** 2022-07-06

**Authors:** Cindy Sing Bik Ngai, Rita Gill Singh, Le Yao

**Affiliations:** 1 Department of Chinese and Bilingual Studies Hong Kong Polytechnic University Kowloon Hong Kong; 2 Language Centre Hong Kong Baptist University Kowloon Hong Kong

**Keywords:** antivaccine misinformation, content themes, writing strategies, COVID-19, virality, social media, content analysis

## Abstract

**Background:**

Vaccines serve an integral role in containing pandemics, yet vaccine hesitancy is prevalent globally. One key reason for this hesitancy is the pervasiveness of misinformation on social media. Although considerable research attention has been drawn to how exposure to misinformation is closely associated with vaccine hesitancy, little scholarly attention has been given to the investigation or robust theorizing of the various content themes pertaining to antivaccine misinformation about COVID-19 and the writing strategies in which these content themes are manifested. Virality of such content on social media exhibited in the form of comments, shares, and reactions has practical implications for COVID-19 vaccine hesitancy.

**Objective:**

We investigated whether there were differences in the content themes and writing strategies used to disseminate antivaccine misinformation about COVID-19 and their impact on virality on social media.

**Methods:**

We constructed an antivaccine misinformation database from major social media platforms during September 2019-August 2021 to examine how misinformation exhibited in the form of content themes and how these themes manifested in writing were associated with virality in terms of likes, comments, and shares. Antivaccine misinformation was retrieved from two globally leading and widely cited fake news databases, COVID Global Misinformation Dashboard and International Fact-Checking Network Corona Virus Facts Alliance Database, which aim to track and debunk COVID-19 misinformation. We primarily focused on 140 Facebook posts, since most antivaccine misinformation posts on COVID-19 were found on Facebook. We then employed quantitative content analysis to examine the content themes (ie, safety concerns, conspiracy theories, efficacy concerns) and manifestation strategies of misinformation (ie, mimicking of news and scientific reports in terms of the format and language features, use of a conversational style, use of amplification) in these posts and their association with virality of misinformation in the form of likes, comments, and shares.

**Results:**

Our study revealed that safety concern was the most prominent content theme and a negative predictor of likes and shares. Regarding the writing strategies manifested in content themes, a conversational style and mimicking of news and scientific reports via the format and language features were frequently employed in COVID-19 antivaccine misinformation, with the latter being a positive predictor of likes.

**Conclusions:**

This study contributes to a richer research-informed understanding of which concerns about content theme and manifestation strategy need to be countered on antivaccine misinformation circulating on social media so that accurate information on COVID-19 vaccines can be disseminated to the public, ultimately reducing vaccine hesitancy. The liking of COVID-19 antivaccine posts that employ language features to mimic news or scientific reports is perturbing since a large audience can be reached on social media, potentially exacerbating the spread of misinformation and hampering global efforts to combat the virus.

## Introduction

### Background

Although vaccines are safe and effective in preventing life-threatening diseases, vaccine hesitancy is still prevalent globally [1,2]. Vaccine hesitancy refers to a delay in acceptance or refusal to vaccinate despite the availability of vaccines [3]. Vaccine hesitancy hovers along the continuum between the two extreme poles of high vaccine demand and vaccine refusal [4]. Vaccine hesitancy is viewed as one of the top 10 threats to global health [5] as it can compromise the herd immunity required to contain pandemics and lead to a greater transmission of the virus [6,7], particularly hampering efforts to curtail the COVID-19 pandemic.

Many complex reasons relating to sociodemographic factors and public trust account for vaccine hesitancy [6,8,9], with misinformation being the main factor [1,7,10]. The World Health Organization (WHO) has used the term “infodemic” to refer to “the rapid spread of misleading or fabricated news” [11]. The topic of vaccination is subject to misinformation [12], particularly for newer vaccines [8], and the proliferation of misinformation, which has fueled fear about vaccine safety and its side effects, is regarded as the main cause of vaccine hesitancy [13,14]. Considerable research has found evidence of how misinformation about vaccines has led to lower vaccine intentions and uptakes [9,12,15-17].

It is worth explaining the distinctions between various terms that refer to misinformation. After the US presidential election in 2016, “fake news” as a phrase has gained considerable attention [18]. Fake news overlaps with other types of misleading information such as misinformation and disinformation. They can be distinguished primarily by the intent and mode of spread [18]. Misinformation is defined as “any health-related claim of fact that is (…) false or inaccurate due to a lack of scientific evidence” [19] and is shared by someone unwittingly without an intention to cause harm [20]. Misinformation specifically refers to claims that draw conclusions using incomplete or wrong information [21]. Conversely, disinformation refers to someone who deliberately creates and disseminates false information with an intention to cause harm [20]. While fake news has received substantial attention, it is difficult to define and has been used by some political groups to undermine certain news media [22]. Drawing on a previous study investigating health-related misinformation on social media [18], in this paper, we use the term *misinformation* as an umbrella term to refer to false or inaccurate health-related information about COVID-19 vaccines, irrespective of the intent, which is difficult to determine.

### Impact of Misinformation on Social Media

Social media, recognized for its openness and participatory nature [23,24], is a common source to receive health information [25], share information about vaccines [16], and receive emotional support in crises [26]. Users can enhance their knowledge about a new disease, its transmission, and preventive measures [27]. However, at the same time, social media can be a source of widespread propagation of fake news, as users can post misinformed claims about vaccines, amplifying concerns about vaccines and resulting in increased vaccine hesitancy [12,15,16,28,29]. This poses a threat to public health and disrupts efforts to prevent disease via vaccines globally [5,29-31]. A recent study [32] highlighted that people exposed to vaccine information on social media have a higher proclivity to be misinformed and have vaccine hesitancy.

In the context of COVID-19, increased usage of social media is seen [33,34] alongside an increased amount of misinformation, negatively affecting public health [35]. Being exposed to COVID-19 information on social media has been linked to higher susceptibility to misinformation [36], resonating with the literature showing that the public is likely to be exposed to misinformation on social media [37]. A few factors have contributed to the rising influence of misinformation on COVID-19 vaccines on social media. These include the notion that lockdowns resulting from COVID-19 in many countries rendered people to have more time to access social media [34], thereby increasing the likelihood of exposure to misinformation. Moreover, since such news tends to be amusing and novel, it encouraged sharing behavior [38]. This has been evidenced in an observational study conducted in Italy, where 2000 articles posted on COVID-19 were analyzed, with articles containing misinformation shared 2 million times, constituting 78% of the total shares of all articles [39]. Another factor relates to many social media sites (eg, Twitter) adopting a strict limit on characters, meaning that the information presented may not be contextualized, making it misleading or incomplete [12]. In the early phase of the COVID-19 pandemic, social media companies did not adopt timely actions against misinformation on their sites [20].

Prior studies have documented evidence of misinformation about COVID-19 on social media [12,40,41]. Some typical examples are that more antivaccine messages were evident on Twitter than provaccine messages [12], while viewers were likely to encounter antivaccine videos on YouTube [40]. A poll conducted by Ofcom showed that 46% of British people reported having been exposed to misinformation about COVID-19, and of those who were exposed, approximately 66% reported watching these sources every day [42], thereby accelerating beliefs in misinformation because of repeated exposure [43]. Several studies have shown that the exposure to misinformation is closely associated with vaccine hesitancy (eg, [9,12,15]).

While there is a large body of research on the association between antivaccine misinformation and vaccine hesitancy [6,7,9,25,44,45], the specific content themes of discussion regarding antivaccine misinformation about COVID-19, how these content themes are manifested in writing through the use of certain writing strategies, and how these themes affect virality on social media warrant examination. Previous studies have so far mainly focused on content themes relating to misinformation and their association with vaccine hesitancy via surveys and experimental studies without considering a range of writing strategies employed to disseminate these messages and the use of social media (eg, [6,7,9,45]). Recently, researchers have investigated these aspects on Facebook and Twitter [25,44]; however, insufficient attention has been paid to *how* content is manifested in writing via the use of certain strategies [12,46-48]. Studies often overlook the use of such strategies in antivaccine misinformation. Given that the public creates and extracts meanings from social media posts [49], the analysis of language, such as determining how content themes are manifested through the use of writing strategies, is critical in gaining a comprehensive understanding of the misinformation that is shared on social media.

Additionally, only a handful of studies have investigated the impact of misinformation on vaccine hesitancy as exhibited in virality in the form of comments, reactions, and shares on social media (eg, [12,17,46,50-53]). Specifically, one study focused on both pro- and antivaccine themes on Twitter from 2014 to 2017, noting that safety concerns and conspiracy theories were the most prevalent themes, and these themes were associated with sentiment-based opinions [12]. Another study analyzed negative and positive comments to Facebook posts on vaccine hesitancy related to human papillomavirus (HPV) in South Africa [17]. Other studies focused on HPV on Twitter and YouTube [50,51]. However, scarce attention has been accorded to the impact of misinformation related to COVID-19 vaccines on virality on social media. More importantly, the ways in which the content themes are manifested in the writing strategies that social media users employ [54] may facilitate the spread of misinformation [55], since the public comprehends the provided information based on their discursive resources [49]. This study is therefore deemed a worthwhile endeavor to undertake.

### Developing an Integrated Framework of Antivaccine Misinformation on COVID-19

#### Strategy Overview

Based on the gaps identified above, in that insufficient attention has been paid to how content themes on antivaccine misinformation alongside how such themes are manifested in writing are associated with virality of misinformation on social media, particularly in the context of COVID-19, the need for this study was evident. Most studies have examined content themes on COVID-19 vaccine hesitancy and misinformation [25,44] without considering how these themes are manifested in writing through the use of writing strategies. Kata’s [47] seminal work on a content analysis of antivaccination websites revealed not only the presence of a variety of content themes but also the writing strategies on these sites, namely amplification strategies, using credible sources untruthfully, misrepresenting facts and statistics, and personal testimonies. Her work has been drawn upon by scholars, which will be elaborated in the next section. A review of the literature (eg, [12,46,47]) indicates that certain writing strategies are used to make misinformation more credible. These strategies comprise: (1) mimicking the language features and format of mainstream news media and scientific reports, (2) using a conversational style such as a personal and informal tone of writing, and (3) employing amplification or exaggeration. Drawing on the above, we developed a framework by integrating two key dimensions, content themes on antivaccine misinformation and the writing strategies used to convey this information, to investigate how these aspects are associated with the virality of misinformation on social media. The findings can inform public health communication efforts with respect to how the public responds to these themes, and consequently offer targeted interventions from social media platform providers, health organizations, and governments to reduce COVID-19 vaccine hesitancy. In the following, we explain these two key dimensions of our study: content themes and writing strategies.

#### Content Themes

##### Theme Development

Following an extensive survey of the published literature, although vaccine hesitancy is influenced by many factors in different historical, political, and sociocultural contexts [8,56], the main attributional factors in terms of antivaccine misinformation content themes appear to be safety, conspiracy, and efficacy [12,14,25,47]. Therefore, we incorporated these content themes into our proposed framework.

##### Safety Concern

Studies show that vaccine safety concern is an important factor causing vaccine hesitancy [12,38,47,57]. Antivaccine safety concern is defined as content that discredits the safety of vaccines and may include notions that vaccines cause harm or death without providing immunity [12,47,58]. This concern is amplified by misinformation spread on social media, in particular that COVID-19 vaccines were developed very quickly, and are therefore unsafe and that all of the side effects have yet to be investigated [38]. In a recent study of parents in Australia, approximately 24% of participants were reluctant or not sure of getting a COVID-19 vaccine and of these, 89% had concerns about vaccine safety [59]. In high-income countries with effective vaccination programs, the fear of safety risks of vaccines far surpassed the fear of the diseases that vaccines prevent [60].

##### Conspiracy Theories

Conspiracy theories are associated with exposure to misinformation on social media [14,61]. This content theme presents specific conspiracy theories, which may encompass stories of fake claims of microchips and poison found in vaccines; fraud; collusion between pharmaceutical companies, governments, and doctors; and pharmaceutical companies manipulating data on vaccine efficacy to make huge profits [12,14,25,47]. A growing number of studies have shown that conspiracy beliefs are associated with vaccine hesitancy and uptake [6,7]. In the United States, beliefs in COVID-19 conspiracy theories were negatively related to the perceived safety of vaccinations and willingness to get vaccinated [45]. The explanation for this is the reduced perceptions of the threat and concerns about safety [62]. Conspiracy beliefs have a widespread influence and discourage vaccine uptake because they are difficult to counter, and are linked to a propensity to reject information from science experts [63]. Beliefs in one conspiracy theory are often tied to beliefs in others, indicating that the public is more likely to trust these beliefs irrespective of their content [64].

##### Efficacy Concerns

This content theme presents vaccines as ineffective and unnecessary, emphasizing that they are unsuccessful and that an increased incidence in the disease is seen after vaccination [12,47]. For example, instead of preventing disease, it is believed that one is more susceptible to getting COVID-19 from the vaccine. In one instance, statistics were cited showing that most people contracting vaccine-preventable diseases (VPDs) were those who had been vaccinated, indicating that vaccination is ineffective [47]. In the study on parents in Australia cited above, approximately 24% of participants were reluctant to get a COVID-19 vaccine and of these, 89% had concerns about vaccine efficacy, believing that the vaccine was unnecessary [59].

The above studies on content themes generated the first research question (RQ1): Are there any differences in the content themes disseminated in COVID-19 antivaccine misinformation on social media?

#### Writing Strategies

##### Categorization

Kata’s [47] pioneering study on content themes and writing strategies employed on antivaccine websites has been drawn on by several researchers. Given the prevalent use of social media in the last 10 years, such strategies might have undergone some changes. Jamison et al [12] built upon Kata’s [47] typology in their analysis of the types of vaccine misinformation on Twitter from 2014 to 2017 by using a data set of 1.8 million tweets. They acknowledged that most of Kata’s [47] content themes and writing strategies were still relevant, but they also observed that amplification strategies such as hashtags to promote content and @messages to high-profile people and organizations to gain attention were commonly seen [12]. Further, they found that antivaccine claims were frequently presented on Twitter as truths by mimicking the language of mainstream news or science [12]. The few studies documented on writing strategies used in antivaccine misinformation suggest that such misinformation is typified by a personal and conversational tone, as evidenced by the use of short texts to enhance comprehension by the public [12,48,65,66]. Personal experiences or anecdotes are emphasized to appeal to the public’s emotions [34]. Drawing on these studies, we categorized writing strategies into the following three types.

##### Format and Features of Writing That Mimic the Language of Mainstream News or Science

Antivaccine claims can be posted as legitimate truths by imitating the language of mainstream news or science experts and presenting them in an accessible language to laypeople [12,46]. Previous research has shown that misinformation that is scientific-sounding is related to lower vaccine intentions [9]. In a Twitter study over the period of 2014-2017, some claims were found to be presented as facts by mimicking the language features used by science experts or the news [12]. In another study on 16,768 tweets on Twitter in 2018, statistics were distorted to support antivaccine claims [46]. In a study on antivaccine misinformation on websites in 2010, credible sources were used dishonestly, false conclusions were derived, and statistics were misrepresented [47]. For example, statistics were quoted showing that the majority of people who got VPDs had been vaccinated, demonstrating that vaccination was ineffective; however, statistics on the high number of unvaccinated people who contracted VPDs were not indicated [47]. Drawing on the findings of these studies, we argue that mimicking of mainstream news and scientific reports can be manifested through the use of writing strategies such as explaining actions taken by health institutions/medical experts; quoting from public figures; using jargon and statistics; attributing information to credible-sounding sources, including medical experts/health organizations and scientific studies; and capitalizing all letters of the first word in a sentence/heading.

##### Use of a Conversational Writing Style

Language can be utilized in different ways to express ideas. One way in which this is done is via the use of a conversational/personal tone of voice or a formal/impersonal tone [46,47]. The former notion is more relevant to antivaccine misinformation, as has been shown in studies where antivaccine misinformation is dominated by a conversational and personal tone as well as personal experiences/anecdotes, which induce fear, anxiety, and mistrust [12,48,65,66]. Personal experiences serve an important role in appealing to the public’s emotions by instilling fear and using blame rather than appealing to logic [38,46]. Existing literature suggests that antivaccine messages often adopt a conversational style by using short sentences or texts, sentence fragments, and questions, facilitating the public’s comprehension and making the language accessible to anybody [46,48,67]. Specifically, Italian webpages disseminating squalene-based influenza provaccine information had on average longer words and sentences that reduced their readability, whereas antivaccine webpages were easy to read [48]. Other researchers also found that in comparison to antiinfluenza immunization online messages, the proinfluenza immunization messages were more difficult to read due to their formal writing style [67]. Building on these studies, we argue that the conversational style and personal tone of voice is manifested in the use of informal expressions (eg, sentence fragments, questions, contractions, emojis), use of first- and second-person pronouns, author visibility, and sharing of personal experiences.

##### Use of Amplification

Amplification refers to how information can be distorted, amplified, or exaggerated on social media [46,68]. Antivaccine advocates have utilized Facebook and Twitter to disseminate exaggerated claims [12,46]. In a study on vaccine hesitancy on Twitter, it was found that most of the negative tweets on COVID-19 contained a hashtag as opposed to positive and neutral tweets [69]. Similarly, a study showed that antivaccine claims on Twitter in 2018 relied on the use of hashtags [46]. Another example pertains to the link between the measles-mumps-rubella vaccine and autism in the Wakefield study [70], which was retracted in 2010; however, Google Scholar statistics indicated that as of June 26, 2018, the Wakefield study had been cited 1090 times since 2012. It should be noted that some of these citations highlighted the flaws in the study, whereas other studies did not do so, suggesting amplification [68]. Two amplification strategies are considered in this study. The first is the use of hashtags, which are popular on social media; in particular, content on Twitter tends to use a large number of antivaccine hashtags to amplify its messages [12,71]. The second frequently used amplification strategy considered is the use of @messages to celebrities and public figures to seek their attention [12].

Following this, the second research question (RQ2) posed is: Are there any differences in the writing strategies manifested in COVID-19 antivaccine misinformation on social media?

#### Virality of Social Media Posts

It is vital to examine the synergistic effect of antivaccine misinformation as exhibited in content and writing strategies on the virality of misinformation on social media. Virality is a term referring to the wide reach or attention of a social media activity or post [72,73]. Viral posts can reach a large audience [74], having far-reaching consequences. The literature has shown that virality can be observed from indicators such as likes, shares, favorites, and retweets on Twitter and Facebook [73,75]. Since our study focused on Facebook, we used the indicators likes, shares, and comments. Social media users use “likes” to indicate their interest in and attention to a topic [76], whereas a “share” is an indicator of user recommendation due to its extended communication [77]. A “comment” offers a platform for discussion since it requires the online user to reply to the post [78].

A rise in likes or shares for a post results in virality [73]. Some content themes attract substantial attention and become viral, increasing the likelihood that they will be shared with the public [79]. Previous studies have found mixed results on the type of content that is associated with virality. Positive and emotionally written articles that evoked strong emotions such as anger, and those with high practical value were more likely to be shared [80]. Yet, in another study, emotional posts had a negative relationship with virality on Twitter, Facebook, and Google, while posts with high practical utility were less often shared on Facebook [79]. Hansen et al [81] found that negative-news Tweets were more often retweeted. Additionally, antivaccine videos on HPV on YouTube led to more likes than provaccine videos [82,83].

Based on this previous work, the aim of this study was to examine the impact of antivaccine misinformation about COVID-19 on virality as exhibited in comments, shares, and likes.

The last research question (RQ) was thus derived as follows: What is the association between the content themes on COVID-19 antivaccine misinformation and the writing strategies used for the dissemination of this news on the virality of misinformation as exhibited in likes, comments, and shares?

## Methods

### Data Collection and Sample Period

We first constructed a database containing antivaccine misinformation circulating on social media for the examination of how COVID-19 misinformation exhibited in the form of content themes and manifested in writing strategies was associated with virality on social media. Antivaccine misinformation was retrieved from two prominent global fake news databases, International Fact-Checking Network (IFCN) Corona Virus Facts Alliance Database [84] and COVID Global Misinformation Dashboards [85], which aim to combat the infodemic by tracking and debunking COVID-19 misinformation [86]. The former was developed by the Corona Virus Facts Alliance, a committee under Poynter’s IFCN, which covers COVID-19–related misinformation from fact-checkers in over 70 countries and in 43 languages of different text types funded by the Canadian Institutes of Health Research, Compute Canada, and the WHO. The latter is situated under the COVID-19 Misinformation Portal, which was developed and managed by the Social Media Lab at the Ted Rogers School of Management in Toronto. This portal tracks and visualizes coronavirus claims from more than 100 trusted fact-checkers. Both databases were developed by leading institutions and global organizations, having been widely cited in previous studies (eg, [87-90]), thus serving as reliable databases for sourcing data in this study.

To yield vaccine-related misinformation, we manually filtered vaccine-related misinformation using the keyword “vaccine” on the IFCN Corona Virus Facts Alliance Database and COVID Global Misinformation Dashboards from September 15, 2019, to August 16, 2021. In total, 2369 and 2298 fact-checked articles on “vaccine” were yielded from these two databases, respectively. Because these databases mainly publish review articles providing fact-checked reports on misinformation collected from multiple media sources (eg, online news, social media posts) in various languages, we trained a postgraduate student majoring in communication studies to carefully scrutinize 4667 vaccine review articles in these databases for retrieving the original links to the antivaccine misinformation on social media (eg, Facebook, Twitter, Instagram), although most links to the original sources were unavailable (ie, removed or deleted after being fact-checked).

To harvest the antivaccine misinformation on social media platforms that was available and comprehensible to social media users globally, two postgraduate students in communication studies were trained to manually visit and review 4667 fact-checked articles, as well as to check and retrieve the available original or archived posts in English and their related viral responses (likes, comments, and shares) on social media platforms. Finally, the trained students combined the yielded items from the two databases by removing the overlapping antivaccine fake news. YouTube was not included in the review process, since most original antivaccine misinformation videos had been removed.

In total, 350 posts containing misinformation on Facebook (n=285, 81.4%), Instagram (n=61, 17.4%), and Twitter (n=3, 0.8%) were yielded. As some posts only consisted of images and videos, and some were kept in an archive and thus some of their viral responses were unavailable, we filtered and retained posts that contained text messages (which included text-only posts and posts with image/video and text) and posts that generated virality in the form of likes, comments, and shares. Because most text-based posts were found in Facebook, the most frequently used social media platform that has gained more active users in recent years [91,92], we decided to focus our text-based analysis on only Facebook posts in this study. Subsequently, we managed to capture 140 posts with all three indicators of virality (likes, comments, and shares) for further examination.

### Content Analysis and Coding Scheme

Once our database had been constructed, we employed quantitative content analysis [34], a research method allowing researchers to conduct quantitative analysis on media messages in a scientific manner [93] to generate generalizable predictions [94] and draw conclusions [30,95]. Additionally, content analysis targets the context in which the occurrences of words, phrases, signs, and sentences are recorded, while offering an in-depth understanding [96]. As such, it is well-suited to a coding operation for a developed framework in media communication [30].

The coding scheme was developed based on the framework proposed in the previous section. Our framework consisted of two dimensions: the first dimension examined the content themes disseminated in misinformation posts and the second dimension focused on the writing strategies manifested in the content themes. The three subdimensions in the content themes included safety concerns (ie, posts that discredited the safety of vaccines), conspiracy theories (ie, posts that highlighted specific conspiracy theories), and efficacy concerns (ie, posts that advocated vaccines as ineffective and unnecessary). Three subdimensions were included in the writing strategies, namely mimicking the format or language features (ie, posts that mimicked the format and language features typical of real news or scientific reports), using a conversational style (ie, posts that were characterized by a conversational style and an informal, personal tone of voice), and using amplification (ie, posts that exaggerated the message by using hashtags and @messages to celebrities and public figures). Table 1 provides a description of the six subdimensions and their references.

Our examination of the data revealed that a post could contain multiple content themes to discredit vaccination. To minimize the loss of information, we coded the presence or absence of the subdimensions on a sentence basis [95]. For example, we coded the dominant subdimension in the content themes dimension to capture all content themes that were present when coding such posts. Textbox 1 shows a representative in-post text extracted from the database. This text first questions vaccine efficacy, suggesting that the vaccine is unnecessary and then continues to claim that the vaccine is unsafe due to its fatal side effects. Thus, the first and second sentence were coded as “efficacy concerns” and “safety concerns,” respectively.

Likewise, a post could employ more than one writing strategy. The first sentence in Textbox 2 mimicked a typical structure of fact-based news (eg, capitalizing all letters of the first word, describing actions of prominent staff from health institutions, using statistics), and was thus coded as format and language features that mimicked news media or scientific reports. The following three sentences adopted a different strategy, indicating a conversational style/personal tone of voice (eg, using sentence fragments; first-, second-, and third-person pronouns; contractions; and questions). Therefore, it was coded as a “conversational style.”

Since the post length varied from 1 to 17 sentences in the collected posts, we decided to normalize the data by dividing the number of sentences coded in each subdimension by the total number of sentences in each post.

**Table 1:** Description of the six subdimensions and their references.

Dimensions and subdimensions		Descriptions		References
**Content themes**				
	Safety	Posts that discredit the safety of vaccines (eg, vaccines can cause harm or death)	[12,38,47,58,59]	
	Conspiracy	Posts that highlight specific conspiracy theories (eg, stories of fake claims of microchips found in vaccines; fraud; collusion between pharmaceutical companies, governments, and doctors; and pharmaceutical companies manipulating data to reap huge profits)	[6,12,14,25,45,47,61,63,64,97,98]	
	Efficacy	Posts that advocate vaccines as ineffective and unnecessary, emphasizing that they are unsuccessful and an increased incidence in the disease is seen after vaccination	[12,47,59]	
**Writing strategies**				
	Format and language features mimicking news or scientific reports	Posts that mimic the format and other features typical of real news or scientific reports. This is exhibited in the following ways: capitalizing all letters of the first word (eg, BREAKING, JUST IN); describing actions and quoting sentences from public figures; attributing information to credible-sounding sources, including medical experts, doctors/nurses, scientific studies, legal documents; using jargon, terminology, and/or statistics	[9,12,46,47,99]	
	Conversational style	Posts that are characterized by a conversational style or an informal, personal tone of voice. This is exhibited in: first- or second-person address form (eg, we should listen, you must act...); author visibility such as sharing personal experiences and feelings; and use of informal expressions (eg, using sentence fragments, questions, contractions, emojis, swear words)	[38,46,47]	
	Amplification	Strategies used to amplify or exaggerate the message. This is exhibited in the use of hashtags and @messages to celebrities and public figures	[12,46,68,70,71]

**Content themes present within one post.**

- Are they really telling us that all 7,800,000,000 people in the world need to be vaccinated for a ‘virus’ that does not kill 99.99% of us?? …
- Reactions to the vaccine would kill more than the ‘virus’.

**Writing strategies present within one post.**

- NEW: About 40-50% of CDC, FDA employees are refusing the COVID-19 vaccine according to Fauci, Marks — Breaking911 (@Breaking911) May 14, 2021.
- Double standards?
- What do they know they aren’t telling us?
- and You wonder why there’s no trust???????

### Intercoder Reliability

Coding was performed by a doctoral student and a postgraduate student majoring in communication studies. Training was provided to both students by the first author before conducting the coding exercise, and the coders were invited to cocode 50 posts (ie, 30% of the total posts) during the training. The measure of intercoder reliability was calculated using the Cohen κ metric. The average Cohen κ of coded items was greater than 0.85, indicating almost perfect agreement [100].

### Statistical Analyses

To fully reveal the weighting of specific content themes and writing strategies in each post, the counted number of sentences in each variable was divided by the total number of sentences in the corresponding post. We then employed analysis of variance (ANOVA) and the posthoc Tukey test to detect and compare the use of different content themes (RQ1) and writing strategies (RQ2) in antivaccine misinformation, since a previous study confirmed the robustness and validity of ANOVA in testing the differences between independent variables, even if the normality assumption is violated [101].

In answering the last research question on the interaction between the content themes on antivaccine misinformation and the strategies used for the dissemination of this news on virality as exhibited in comments, reactions, and shares (RQ3), we first employed Poisson regression, a count regression model, in SPSS [102]. It was found that our data violated the assumption in Poisson regression due to an overdispersion of outcome variables, which is common in real-world data sets [103]. We therefore followed the common practice of replacing Poisson regression with negative binomial regression (NB2) to improve the goodness of fit, especially the Akaike information criterion and Bayesian information criterion [103]. NB2 is effective in fitting various types of data in communication and technical research, and is a more general model that relaxes the strong assumption that the underlying rate of the outcome is the same for each included participant [104].

## Results

**Figure 1:**
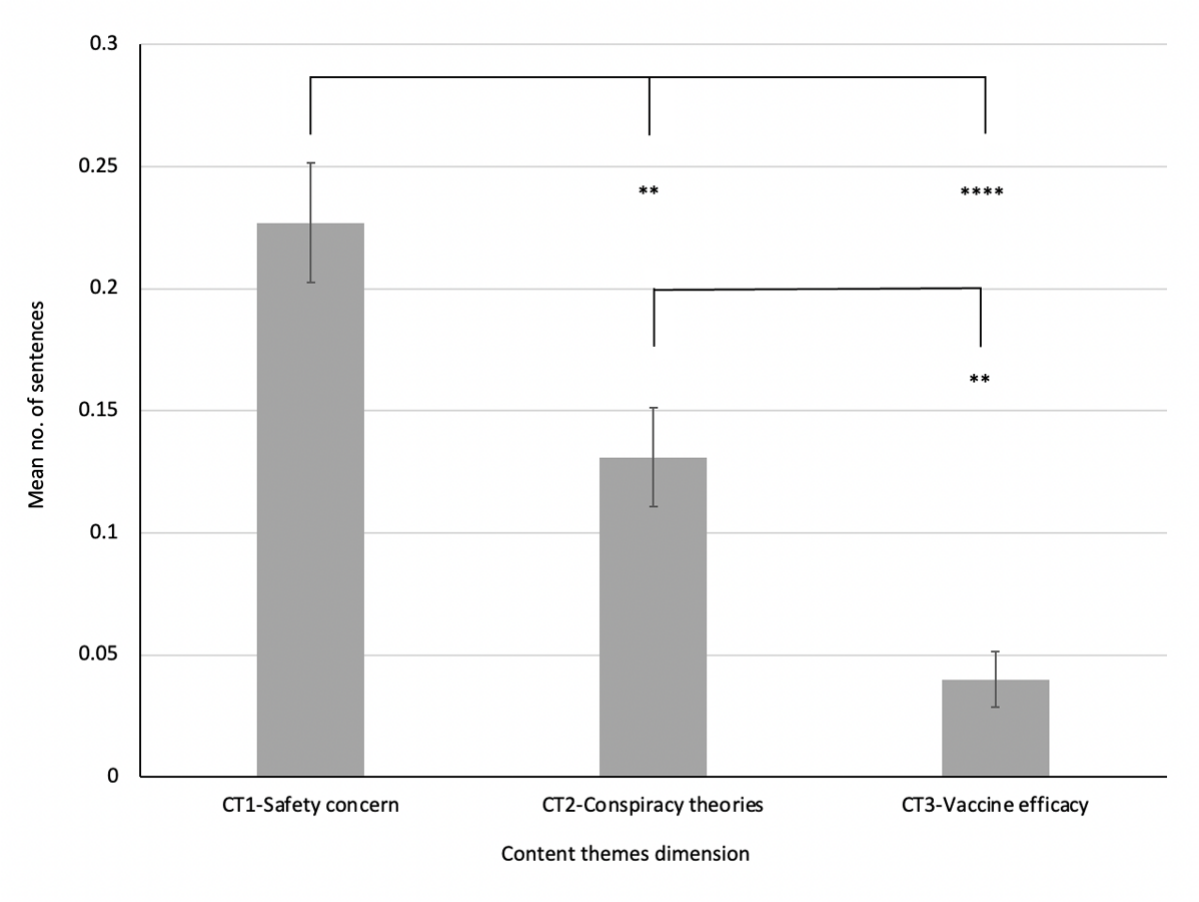
Mean count of sentences disseminating content themes (CT) in COVID-19 vaccine misinformation posts on Facebook.

In response to RQ1, inquiring into whether there was any difference in the content themes disseminated in antivaccine misinformation on social media, the findings showed that safety concern was the most prominent theme, followed by conspiracy theories and efficacy (Table 2). The ANOVA results confirmed a significant difference in the content themes communicated in antivaccine misinformation (*F*_2,417_=21.20, *P*<.001). The posthoc Tukey test indicated that the content theme safety concern was significantly higher than conspiracy theories (*P*=.003) and efficacy (*P*<.001), whereas conspiracy theories was also significantly higher than efficacy (*P*=.005). Table 2 provides the descriptive statistics on the examination of content themes disseminated in COVID-19 vaccine misinformation posts on Facebook and Figure 1 displays the mean count of sentences disseminating content themes in COVID-19 vaccine misinformation posts on Facebook.

**Figure 2:**
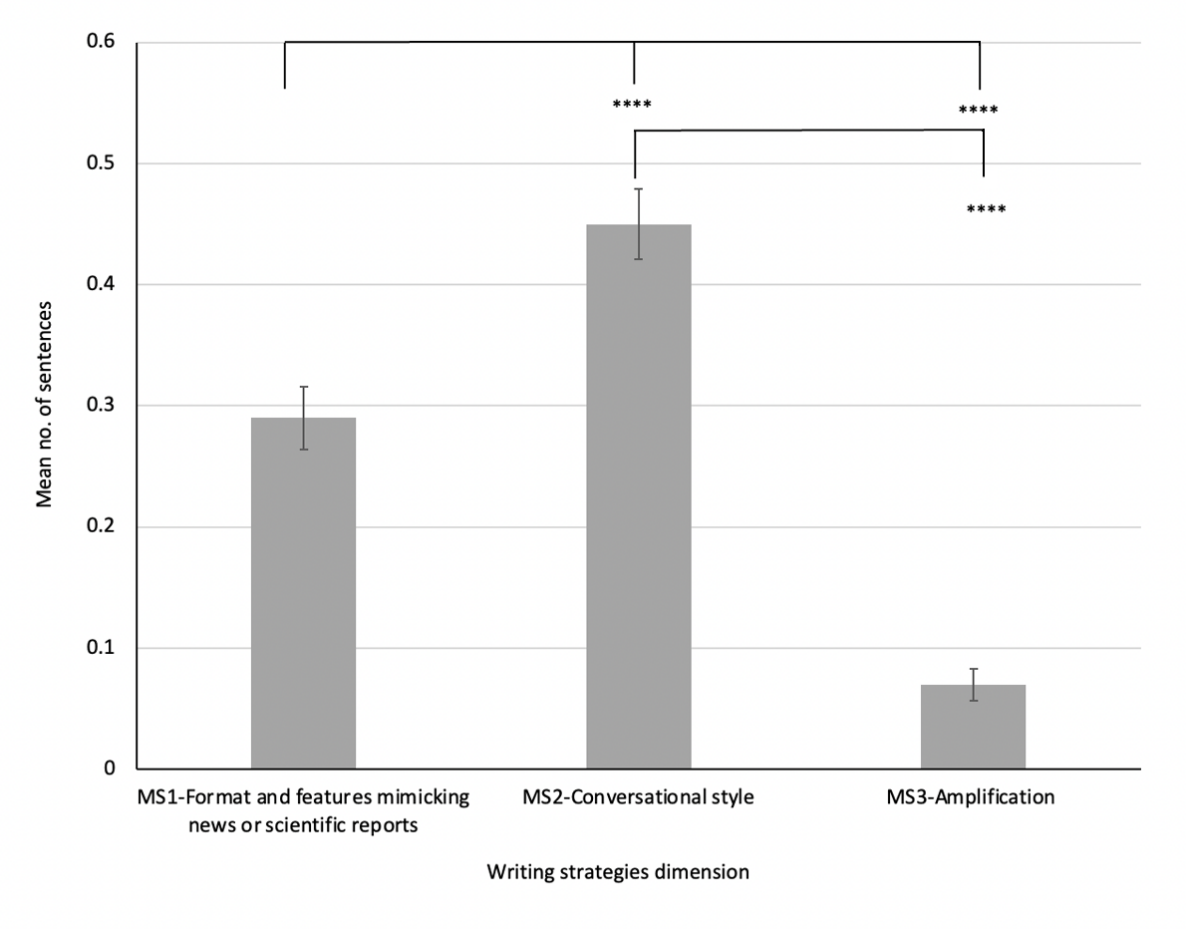
Mean count of sentences employing writing strategies in COVID-19 vaccine misinformation (MS) posts on Facebook.

Regarding RQ2, we investigated if there was any difference in the writing strategies employed to disseminate antivaccine misinformation. Our findings showed that conversational style was the most frequently used strategy, followed by format or language features mimicking news or scientific reports and amplification (Table 3). The ANOVA results revealed that there was a significant difference among the use of strategies (*F*_2,417_=61.34, *P*<.001). The posthoc Tukey test confirmed that the conversational style strategy was significantly higher than format or language features mimicking news or scientific reports (*P*<.001) and amplification (*P*<.001), while format or language features mimicking news or scientific reports was also significantly higher than amplification (*P*<.001). See Table 3 for the descriptive statistics on the examination of writing strategies employed in COVID-19 vaccine misinformation posts on Facebook and Figure 2 for the mean count of sentences employing writing strategies in COVID-19 vaccine misinformation posts on Facebook.

Concerning RQ3, examining if there was any association between the content themes on antivaccine misinformation and the strategies used for the dissemination of this news on virality as exhibited in likes, comments, and shares, the NB2 results indicated that safety concern was a significant negative predictor of the number of likes and shares (Table 4). The odds ratio showed that for every extra sentence disseminating safety concerns, there was a decrease of 0.05 the number of likes and 0.30 the number of shares. By contrast, format or language features mimicking news or scientific reports was a strong positive predictor of the number of likes (Table 4). The odds ratio indicated that for every extra sentence utilizing format or language features mimicking news or science, there was an increase of 7.55 number of likes (Table 4).

The Omnibus test of NB2 showed significance with likes (*P*<.0001) and shares (*P*=.05) as a dependent variable, but not with comments as a dependent variable (*P*=.07).

**Table 2:** Descriptive statistics on the examination of content themes disseminated in COVID-19 vaccine misinformation posts on Facebook.

Content theme	Number of posts	Mean (SD)
Safety concern	140	0.23 (0.30)
Conspiracy theories	140	0.13 (0.25)
Vaccine efficacy	140	0.04 (0.14)
Total	420	0.13 (0.25)

**Table 3:** Descriptive statistics on the examination of writing strategies employed in COVID-19 vaccine misinformation posts on Facebook.

Writing strategies	Number of posts	Mean (SD)
Format or language features mimicking news or scientific reports	140	0.29 (0.32)
Conversational style	140	0.45 (0.36)
Amplification	140	0.07 (0.16)
Total	420	0.27 (0.33)

**Table 4:** Identification of positive and negative predictors of the numbers of likes, comments, and shares using a negative binomial regression model.

Variables		Likes				Comments			Shares		
		β (SE)	95% CI	*P* value	β (SE)		95% CI	*P* value	β (SE)	95% CI	*P* value
**Content themes**											
	Safety concern	–3.04 (0.52)	–4.07 to –2.02	<.001	–1.52 (.48)		–2.45 to –.59	.001	–1.19 (.55)	–2.27 to –.12	.03
	Conspiracy theories	.35 (.58)	–.79 to 1.49	.55	.06 (.51)		–.94 to 1.06	.90	.53 (.59)	–.63 to 1.70	.37
	Efficacy	.22 (.83)	–1.40 to 1.84	.79	–.02 (.80)		–1.59 to 1.55	.98	–.32 (.89)	–2.06 to 1.42	.72
**Writing strategies**											
	Format or language features mimicking news or scientific reports	2.02 (.93)	.19 to 3.85	.03	–.10 (.79)		–1.66 to 1.46	.90	–.20 (.75)	–1.68 to 1.28	.79
	Conversational style	.23 (.74)	–1.21 to 1.68	.75	.23 (.69)		–1.12 to 1.58	.74	.26 (.73)	–1.18 to 1.70	.72
	Amplification	.51 (1.16)	–1.76 to 2.77	.66	–.35 (1.03)		–2.36 to 1.67	.74	.70 (1.22)	–1.70 to 3.10	.57

## Discussion

### Principal Findings

The results showed that the most common content themes disseminated in COVID-19 antivaccine misinformation on Facebook were safety concerns, followed by conspiracy theories, which is consistent with previous studies [12,14,38,47]. A noteworthy point is the association between the content themes and virality of misinformation. Safety concern was a strong negative predictor of the number of likes and shares, although it was the most frequently used content theme. This could be attributed to the continued efforts made by governments and health organizations to emphasize the safety of COVID-19 vaccines and the growing threat of COVID-19 (eg, [105-107]). The public is therefore more likely to identify the misleading information disseminated in the posts, and less willing to like or share them when they have learnt more about the safety of vaccines. Earlier studies have shown that users are more likely to share content that has a high quality or practical value [80,108], which may also explain why the public was less willing to share this information.

With respect to the writing strategies manifested in the content themes, the results revealed that a conversational style as well as format and language features that mimicked news media and scientific reports were frequently used to spread antivaccine misinformation. This finding resonates with the literature about antivaccine information, which is typified by a personal, conversational, and negative tone [48,65,66], and a prior study showing that antivaccine claims were presented as facts by imitating the language of science and news on Twitter, leading to a high number of retweets [12]. Given that language can be utilized for different purposes [12,46,49,55], the antivaccine news posts capitalized on these distinct features of language to achieve their purpose of disseminating misinformation. Some prior studies have investigated strategies such as emotional appeal and amplification to disseminate antivaccine news [12,46,47]; however, studies on these aspects relating to COVID-19 on social media are lacking. Therefore, our findings add to the body of knowledge of how content themes are manifested in writing strategies to disseminate COVID-19 antivaccine misinformation.

Our results also confirmed that posts relying on format and language features that mimicked news media and scientific reports were strong positive predictors of likes. These posts might have looked authentic and appealing, thus encouraging liking. It is interesting that while the posts promoted liking, they were not associated with shares, possibly due to the negative information contained in them as well as uncertainty of the source of information, which might have made users hesitant to virally share the information. Facebook had a total of 2.91 billion monthly active users from October to December 2021 [91]; thus, the far-reaching effects of even liking antivaccine posts about COVID-19 should not be downplayed. These liked posts may exacerbate the extent of antivaccine misinformation disseminated on social media, potentially hampering efforts to prevent diseases via vaccines [5,32]. Our novel findings regarding the relationship between virality and the content themes and writing strategies used provide important insights for counteracting COVID-19 antivaccine misinformation.

### Implications, Limitations, and Future Directions

This study contributes to the understanding of which content themes and writing strategies manifested in the themes led to virality of COVID-19 antivaccine misinformation on social media, and adds to the literature on this development subject. By constructing a database of antivaccine misinformation on COVID-19 circulating on social media from two globally leading and widely cited fake news databases for the examination of COVID-19 misinformation exhibited in the form of content themes and writing strategies and their association with virality, we found that posts on safety concerns were the most frequently occurring topic, and this content theme was manifested in writing through the use of a conversational style and format and language features that mimicked news and scientific reports. Additionally, the latter was associated with virality in the form of likes.

Our study thus provides insights into which content themes and manifestation strategies were associated with virality, and could be explored further to counter the impact of antivaccine misinformation. Since vaccine safety predicts vaccine intention, as found in other studies, and safety concern is the most frequently seen content theme susceptible to misinformation [109,110], the importance of countering misinformation on COVID-19 vaccines to increase public acceptance is confirmed. To do this successfully, systematic monitoring of the antivaccine misinformation circulating on social media has to be undertaken. This can be achieved by extracting misleading news posts related to safety and debunking the claims mentioned in these posts, especially those that adopt a conversational style and imitate real news or scientific reports. To discern real news and misinformation, social media platforms or fact-checkers should focus not only on the content but also how it is conveyed by paying more attention to the writing strategies used in such posts. It would be prudent for social media platform providers, governments, researchers, and health organizations to be provided with an updated summary of antivaccine misinformation circulating on social media to help them counter antivaccine concerns and provide accurate information about COVID-19 vaccines.

Like any data set, that used in this study has limitations. Since we only collected the antivaccine misinformation posts for 2 years, different time periods of the evolving COVID-19 pandemic should be considered. It should also be noted that antivaccine misinformation is subject to change over time, and thus our study findings should be interpreted with caution. The data on content themes and the writing strategies manifested in the content, and their associations with virality are correlational only. Most importantly, our study did not focus on social media users’ sentiment-based opinions in the form of comments, which differ in valence (ie, negative, positive, neutral) and can reveal more detailed feelings [111]. An analysis of the valence of comments could have revealed more in-depth reasons that contributed to vaccine hesitancy in relation to antivaccine misinformation. Research suggests that emotions may overtake logic, and therefore studies have addressed antivaccine sentiment impacts [13,112]. Our emphasis on COVID-19 content themes and writing strategies to disseminate such themes can be further empirically tested. Popular social media platforms have reached ubiquitous heights, and examination of other information-sharing platforms such as Instagram’s COVID-19 antivaccine misinformation may shed more light on this topic.

### Conclusions

To summarize, this study presents a novel examination of antivaccine misinformation in terms of content themes on COVID-19 and the ways in which these themes were manifested through the use of writing strategies. The key findings are that posts on safety concerns were negatively associated with likes and shares, whereas posts that mimicked the format and language features of news media and scientific reports were associated with likes on Facebook. This possibly suggests that antivaccine misinformation about COVID-19 has been amplified by liking these posts via social media. We do not yet know how far-reaching the impact of antivaccine misinformation has been, although some evidence indicates that misinformation about COVID-19 has had an impact on the public’s vaccine uptake [113], posing a global health challenge. By drawing on this study’s findings and leveraging the power of social media, platform providers, governments, and health organizations can take measures to counter COVID-19 antivaccine misinformation to reduce vaccine hesitancy, which remains pervasive globally.

## Abbreviations

ANOVA: analysis of variance
HPV: human papillomavirus
IFCN: International Fact-Checking Network
NB2: negative binomial regression
RQ: research question
VPD: vaccine-preventable disease
WHO: World Health Organization
